# Supplementary material for: MetaRibo-Seq measures translation in microbiomes
Source: Nat Commun. 2020 Jun 29;11:3268. doi: 10.1038/s41467-020-17081-z (PMC7324362; doi:10.1038/s41467-020-17081-z)
Supplement: Supplementary file 10 — Supplementary Data 7 [file 41467_2020_17081_MOESM10_ESM.zip › File2/Confidence_High_Taxonomy/94963_out.krona.html]

Javascript must be enabled to view this page.

members
magnitude
magnitudeUnassigned
count
unassigned
taxon
rank

94963\_out

7

superkingdom
7
2

7
1239
phylum

7
186801
class

order
7
186802

family
7
186803

genus
1
572511

1
33039

SRS143466\_contig\_number\_32211
species

species

SRS053398\_contig\_number\_22672SRS055017\_contig\_number\_27268SRS103987\_contig\_number\_32679SRS143070\_contig\_number\_12303SRS144603\_contig\_number\_57SRS147271\_contig\_number\_32387
6
39491
